# Effects of experimentally induced fatigue on healthy older adults’ gait: A systematic review

**DOI:** 10.1371/journal.pone.0226939

**Published:** 2019-12-30

**Authors:** Paulo Cezar Rocha dos Santos, Fabio Augusto Barbieri, Inge Zijdewind, Lilian Teresa Bucken Gobbi, Claudine Lamoth, Tibor Hortobágyi

**Affiliations:** 1 Center for Human Movement Sciences, University Medical Center Groningen, University of Groningen, Groningen, The Netherlands; 2 Posture and Gait Studies Laboratory (LEPLO), Graduate Program in Movement Sciences, Institute of Biosciences, São Paulo State University (UNESP), Rio Claro, Brazil; 3 Human Movement Research Laboratory (MOVI-LAB), Graduate Program in Movement Sciences, Department of Physical Education, São Paulo State University (UNESP), Bauru, Brazil; 4 Department of Biomedical Sciences of Cells and Systems, University Medical Center Groningen, University of Groningen, Groningen, The Netherlands; Universidade Federal do Rio Grande do Sul, BRAZIL

## Abstract

**Introduction:**

While fatigue is ubiquitous in old age and visibly interferes with mobility, studies have not yet examined the effects of self-reported fatigue on healthy older adults’ gait. As a model that simulates this daily phenomenon, we systematically reviewed eleven studies that compared the effects of experimentally induced muscle and mental performance fatigability on gait kinematics, variability, kinetics, and muscle activity in healthy older adults.

**Methods:**

We searched for studies in databases (PubMed and Web of Science) using Fatigue, Gait, and Clinical conditions as the main terms and extracted the data only from studies that experimentally induced fatigue by sustained muscle or mental activities in healthy older adults.

**Results:**

Eleven studies were included. After muscle performance fatigability, six of nine studies observed increases in stride length, width, gait velocity (Effect Size [ES] range: 0.30 to 1.22), inter-stride trunk acceleration variability (ES: 2.06), and ankle muscle coactivation during gait (ES: 0.59, n = 1 study). After sustained mental activity, the coefficient of variation of stride outcomes increased (ES: 0.59 to 0.67, n = 1 study) during dual-task but not single-task walking.

**Conclusion:**

Muscle performance fatigability affects spatial and temporal features of gait and, mainly, inter-stride trunk acceleration variability. In contrast, sustained mental activity tends only to affect step variability during dual tasking. A critical and immediate step for future studies is to determine the effects of self-reported fatigue on gait biomechanics and variability in healthy older adults to verify the viability of experimentally induced fatigue as a model for the study of gait adaptability in old age.

## Introduction

Population studies and primary care data show that ~46% of older adults complain about being tired [1–3]. Tiredness is the sensation of exhaustion, a reduction of physical and mental energy, and a diminished interest in the surrounding world. Prolonged physical or mental exertion can reduce motor performance (performance fatigability) [4–6] or reduce the capacity to allocate cognitive resources to perform a task [7] and increase self-reported fatigue (perceived fatigability) [8]. Performing a low-force activity for a prolonged period, such as a long high-paced walk, can lead to a sensation of muscle performance fatigability. Performing a motor task at a high percentage of the available maximal mechanical output, i.e., at a high relative effort, can also lead to muscle performance fatigability, a state that is associated with reduced contractile force and a sub-optimal neural activation of muscles [6,8,9]. The decline in force due to sustained muscle effort can interfere with the quality of motor acts such as carrying an object, maintaining bodily postures, and gait [10–12].

While prolonged low force and short-term high force motor acts can directly reduce motor performance due to impairment in force and muscle activation, demanding mental activities can also create a psychobiological state characterized by a perception of tiredness and a lack of motivation [8,13,14]. Sustaining attention or a mental effort for a prolonged period puts older adults in a fatigued mental state [7,15,16] that slows cognitive processes often quantified by slowed reaction times [13,14,17]. Sustained mental activity is also associated with alteration of cortical brain areas and decreases in neurotransmitter levels [14,18]. Such modifications may impair top-down cognitive control and the execution of motor tasks indirectly, even in the absence of demonstrable muscle weakness [19,20]. Sustained mental activities can also decrease parasympathetic and increase sympathetic activity, reducing motivation and pre-frontal brain activation [7,20].

While both fatigue types are prevalent in old age and visibly interfere with gait, studies have not yet examined the effects of trait of fatigue on healthy older adults’ gait. To minimize interference and maintain gait quality, older adults are expected to adopt strategies that help to compensate for the mal-effects of fatigue on gait. The unanswered question is whether and how those healthy older adults who report no trait of fatigue can adapt their gait when either kind of fatigue is induced by experimental protocols in a laboratory environment. Such paradigms are thought to simulate performance or perceived fatigued states often reported by older adults. It is important because the after-effects of sustained activities can destabilize gait and posture, increasing the risks for slips, trips, and falls [21–24]. The picture emerging from the systematically not yet reviewed studies is that fatigue-free healthy older adults are able somehow to adjust their gait kinematics, kinetics, variability, and muscle activation to states created by performance or perceived fatigue induced in a laboratory environment [25–27]. It seems likely that experimentally induced muscle fatigability by prolonged physical activity affects the generation of mechanical work and power at the ankle, knee, and hip joints during gait [28]. Such changes are reasonable because the cellular mechanisms of fatigue impair voluntary force generation and the neural drive of muscles [4,6,29] that generate torques and powers during gait. Specifically, it is likely that older adults would in compensation for the force loss increase stride width and muscle activity to increase gait stability [25]. Subtler mechanisms could involve increases in the activity of antagonist muscles and distribute effort by recruiting less affected muscles at adjacent joints [28,30,31]. Concerning mental fatigability, we expect that interference with attention, arousal, executive function, mood, and motivation would primarily affect gait variability [32,33]. Indeed, brain areas underlying these cognitive functions are also active during imagined walking [34] and are related to temporal step outcomes and gait variability [35–37]. We thus hypothesized that gait adaptations might be fatigue-type specific. The purpose of this paper was to systematically review studies that compared the effects of experimentally induced muscle and mental performance fatigability on gait kinematics, variability, kinetics, and muscle activity in healthy older adults. A comprehensive review of these adaptations is timely and needed because it would increase our understanding of how old age affects the capacity to adapt gait to sustained muscle or mental activities.

## Methods

We performed a computerized systematic literature search, following PRISMA (S1 Checklist) and Cochrane Handbook for Systematic Reviews guidelines [38,39], in PubMed and Web of Science for the period between January 1987 to August 2019 (last 30 years from the beginning of the search (2017) and updated for the 2 following years). The search consisted of four terms: Term 1 was the population by using the keywords ‘old’, ‘elderly’ and ‘adults’; Term 2 was the intervention ‘Fatigue’ probed with the keywords ‘fatigue’, ‘fatigability’, ‘tiredness’, and its variants (e.g., mental fatigue, physical fatigue, motor fatigue, cognitive fatigue, performance fatigability, and perceived fatigability). Term 3 was the outcomes ‘Gait’ and ‘Walking’ with the outcomes of gait adaptability concerning gait biomechanics, kinetics, kinematics, muscle activity, spatial-temporal parameters, inverse dynamics, gait stability, and gait variability. Term 4 included the exclusion criteria and clinical conditions, such as neurological and orthopaedical diseases. Although the Cochrane Handbook for Systematic Reviews suggest that the ‘NOT’ operator should be avoided as exclusion where possible [39], in our case, exclusion terms were necessary as a search strategy to remove from the initial screening the substantial number of papers in diseased populations. Filters were set to include English language (S1 Table). The PubMed syntax was adapted to the Web of Science search. We also identified studies missed by the search from the list of references of relevant individual papers.

### Eligibility, study selection and exclusion criteria

We used the Population, Intervention, Comparison, Outcome, and Study design as the criterion for inclusion of papers in this review [38]. Population: older human adults. Intervention: fatigue induced by prolonged physical/muscle and mental tasks. Comparison: gait in fatigue and non-fatigued state. Outcomes: gait kinematics (e.g., spatial and temporal stride parameters, joint angle, joint angular, acceleration outcomes), kinetics (e.g., force outcomes as momentum, work and power, ground reaction force), electromyography (e.g., amplitude and temporal parameters used to assess muscle activation). For the analysis of gait variability and stability, we considered the standard deviation, coefficient of variation, and measures of variability regarding gait dynamics, such as RMS, sample and multi-scale entropy methods, detrended fluctuation analysis, and local dynamic stability and margin of stability, respectively. We also considered gait performed under different conditions such as obstructed gait, level surface walking, and treadmill walking. Finally, randomized controlled trials (RCTs), non-randomized controlled trials (nRCTs), and non-randomized non-controlled trials (nRnCTs) were included.

From the initial yield, obtained by combining original articles from electronic databases and targeted searches, titles and abstracts were screened. When a study was potentially eligible and relevant, it was selected for a full-text analysis and then subjected to a quality analysis. Studies that analyzed the effects of fatigue on gait in age groups other than only in older adults were included, but we considered the data only for older adults (over 63 years). When the information was considered insufficient based on title and abstract alone, the full text was analyzed to decide on inclusion.

We had excluded studies that examined running and stair climbing. In addition, studies unrelated to induced fatigue (decline in performance and/or increase in self-reported fatigue) by sustained physical and mental activities or that could not indicate a measurement of induced fatigue, a lack of quantitative gait outcomes and/or a lack of older adults in the sample were excluded at the initial screening of titles and abstracts.

### Quality assessment

Two of the authors (PCRS, FAB) screened candidate papers and worked based on a set of guidelines to improve inter-rater reliability. Both authors analyzed the methodological quality of the included studies by using a quality appraisal tool [40]. This appraisal tool relates to the internal and external validity of the measurement and the generalizability of the results. For each question, ‘1’ is rated when the criterion was met, ‘0.5’ when information is lacking detail or clarity, and ‘0’ if the criterion was missing. A higher total score represents a higher quality of the study. In case of discrepancies between the two authors, a third author (TH) was consulted to make a decision about inclusion.

### Data extraction and analysis

Two of the authors (PCRS, FAB) extracted the papers, independently, and synthesized data in tables and together, both authors checked the tables. In case of an indecision, a third author (TH) was consulted. The data were coded for: number of participants, age, sex, protocol to induce performance fatigability (sit-to-stand, cognitive task, walking test), measurement of fatigue (decline in performance, increase in self-reported fatigue), gait protocol (treadmill, level walking, walking with obstacle crossing), and gait outcomes (kinematic and kinetic data, variability, muscle activity). It was not necessary to contact any authors to get information regarding the included papers. We used Cohen’s *d* to calculate the effect sizes (ES) to quantify whether the magnitude of changes in gait outcomes induced by sustained muscle or mental activity is relevant. ES values of 0.21–0.49 indicate small, 0.50–0.79 indicate medium, and ≥ 0.80 indicate large practical effects [41]. Due to the heterogeneity of the outcomes, lack of consistent results, and the low number of studies that met eligibility criteria, we were unable to perform a meta-analysis.

## Results

### Study characteristics

**Fig 1:**
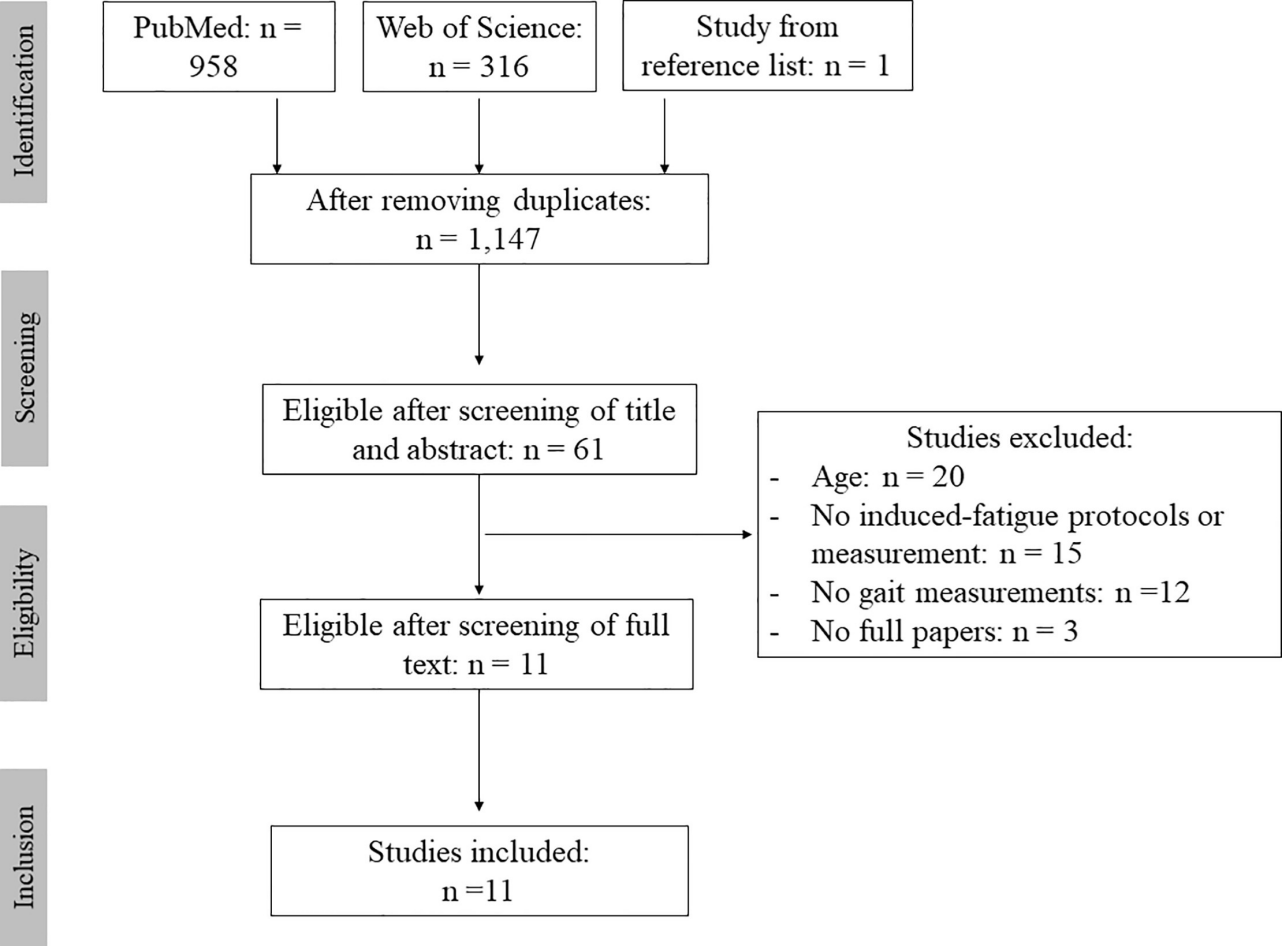
PRISMA flow diagram.

The Pubmed and Web of Science searches yielded 1,274 studies and one study was included from the list of reference [42]. After screening for title, abstract and remove the duplicates, 61 studies were selected for analyses, and, after reading the full text, a final sample size of 11 studies was included in the review (Fig 1). The included studies stated the aims sufficiently, gave an appropriate description of the methods, detailed the outcomes clearly, and provided an interpretation of the key findings (S2 Table).

The current review was based on 249 healthy older adults with a mean age of 71.5 (±4.66) years (Table 1), 92 (37%) females, with normal body composition (body mass index: 26.1±1.94 kg/m^2^). Two studies did not report the subjects’ sex [23,43] (Table 1).

**Table 1:** Description of the characteristics of the papers selected.

	Helbostad et al.[47]	Granacher et al. [27]	Granacher et al.[44]	Hatton et al.[26]	Barbieri et al. [25]	Nagano et al. [23]	Toebes et al. [45]	Arvir et al. [46]	Hamacher et al. [43]	Morrison et al. [21]	Behrens et al. [32]
Participants	44	14	16	30	40	11	10	17	18	30	16
male/female (N)	10/34	14/0	8/8	17/13	40/0	n/m	4/6	5/12	n/m	14/16	6/10
age (yrs)	79.3	67.2	71.3	78.3	69.3	74.2	63.4	73.2	69	69.4	72.2
body index (kg/m^2^)	24.4	25.1	25.2	27.2	26.6	26.2	n/m	24.7	25.5	31.1	25.5
**Fatigue protocol**											
muscle contraction	STS—Knee	IK–Ankle	IK—Knee	STS–Knee	STS–Knee		Squat—Knee UL	Abd—Hip UL			
endurance						6-min fast-Walking			Cycle–Ergometer	Treadmill—Walk	
mental task											Go/ no go test
**Fatigue outcomes**											
parameters	↓ Pace of Mov	↓Force	↓Force	↓Force	↓Force ↑ RPE	↑Heart Rate	↓Force	↑ RPE ↓Prop	↑ RPE	↓Force ↓RT ↑RPE	↓Motiv ↑ Fatigue state

N: sample, n/m: not mentioned, STS: Sit-to-Stand test, IK: Isokinetic, ABD: Abduction, UL: unilateral, Mov: Movement, RPE: Rating of Perceived Exertion, RT: Reaction Time, Prop: Proprioception, Motiv: Motivation.

### Effects of fatigue protocols on fatigue outcomes

The studies used heterogeneous protocols to induce a decline in muscle performance, including repeated muscle contractions (n = 7 studies), knee extension/flexions (n = 5), sit-to-stands (n = 4), endurance (n = 3, treadmill and cycling), isokinetic (n = 2, knee and ankle), hip abductions (n = 1), and prolonged mental tasks (n = 1, go/no go task for 90 min) (Tables 1 and 2). Six, three, and two studies indicated the state of fatigue, respectively, as a decline in voluntary force, inability to perform the movement, and movement slowing (Table 1). The reduction in force ranged between ~10 to ~55% and varied between protocols. The sit-to-stand task, for example, reduced voluntary force by ~9 to 13% [25,26]. The isokinetic protocols considered fatigue as reductions to 50% of the initial maximum voluntary torque [27,44]. Unilateral squats performed until exhaustion reduced knee extension force by 17% [45]. Longer reaction time was also observed post vs. pre sustained endurance protocol [21]. Five studies reported an increase in self-reported fatigue (indicated by the rating of perceived exertion and by fatigue perception) (Tables 1 and 2) [21,25,32,43,46]. Two studies [25,46] indicated that older adults reported near maximal perceived exertion (‘very hard’ to ‘maximum exertion’) after repeated muscle contractions. Two studies [21,43] indicated a high rating of self-reported fatigue in response to endurance protocol, scores ranged from ‘hard’ (15) to ‘maximal exertion’ (20) on the Borg scale. One study [32] determined fatigue as a decrease in motivation by 10%, as examined by wakefulness, mood, and arousal dimension of the Multidimensional Mood Questionnaire (ES range: 0.27 to 0.95) and up 2x of increase in fatigue state assessed by Profile of Mood States (ES: 0.92) following a prolonged period of mental activity. One study [23] also indicated an increase (range: 25 to 35 beats per minute) in heart rate (ES: 0.65) after endurance exercise.

**Table 2:** Study characteristics for included studies.

		Fatigability		Gait		
Study	N–older adults	Protocol	Outcomes	Gait Conditions	Gait Outcomes	Fatigue-related changes (effect sizes)
Helbostad et al. [47]	22—Fatigue Group (FG) 22—Control Group (CG)	Sit-to-stand	↓ time and vertical displacement of movement the sit-to-stand	Overground level walking (LW)	AP, ML and V. Trunk acc. and inter-stride trunk acc var;. SL, SW, and Sp; SL-var and SW-var.	FG vs. CG: ↑ SW (ES: 1.51), ML trunk acc (ES: 1.27), SL Var (ES: 2.61) and ↓ V. (ES: 2.06) and AP (ES: 0.80) inter-stride Trunk acc var
Granacher et al. [27]	14	Isokinetic ankle extension	↓ in ~50% of maximal torque	Perturbation (decelerating) on treadmill walking	Functional reflex activity (FRA) and latency of m. Tibialis Anterior (TA), Latency in TA, EMG activity of the m. Peroneus, Soleus and Vastus Medialis, Coactivity and maximal angular velocity.	↓ FRA in TA (ES: 0.56), ↑ coactivity (ES: 0.58) and maximal angular velocity (ES: 0.64).
Granacher et al. [44]	16	Isokinetic knee extension	↓ in ~50% of maximal torque	LW in single-(ST) and dual-task (DT)	SdL, Gait Sp, DT cost in SdL and gait Sp and the Standard deviation of the SdL in ST and DT conditions	DT: ↑ Gait Sp (ES: 0.55); SdL (ES: 0.45) and ↓ SD of SdL (ES: 095).
Hatton et al. [26]	30	Sit-to-stand	↓ in 9.5% of the peak of force on knee extension	Obstructed walking (OW) with a secondary visual task	Std, Sp of obstacle crossing, Trail and lead limb vertical and horizontal distance to the obstacle, and V. loading rate.	↑ V loading rate of the lead limb (ES: 0.27).
Barbieri et al. [25]	20 –(60–70 years—G60) 20 –(over 70 years—G70)	Sit-to-stand	↓ in ~13% of the peak of force ↑ RPE	LW and OW	SdL, SdD, Sp, and SW (LW and OW). SL, Sd, Sp, Trail (T) and Lead (L) vertical distance to the obstacle (VO).	LW and OW: ↑ SdL / SL (ES: 0.35 / 0.04), SW (ES: 0.36 / 0.19), Sp (ES: 0.65 and 0.31), ↓ SdD/Sd (ES: 0.43 and 0.45). OW: ↑ TVO (ES: 0.1)
Nagano et al. [23]	11	Endurance (treadmill walking)	↑ ~35% in heart rate	Treadmill walking	SL (normalized by limb length), DsT (%) and SW and Minimum Foot Clearance	↑ SL (ES: 0.63), DsT (ES: 0.12), Var SW (ES: n/p) ↓ Minimum Foot Clearance (ES: 0.7)
Toebes et al. [45]	10	Unilateral squat exercise until task failure.	↓ 17.3% Knee extension strength	Unperturbed and perturbed (push the trunk) treadmill walking	3-D LyE of the trunk, trunk vel, and var of trunk vel, time to return to unperturbed gait pattern on stance and swing phase. Deviation of trunk kinematic after perturbation.	↓ Time to return to the unperturbed gait pattern on swing phase (ES: 0.67) and deviation after perturbation (ES: 1.8)
Arvir et al. [46]	17	Unilateral hip abductor	↓ Hip position sense and ↑ RPE	Treadmill walking	SdD means and standard deviations; ML trunk vel; Harmonic Ratio (HR) of ML and AP; Local Divergent Exponents of ML and AP, acceleration and position.	↑ SdD Var (n/p) and ↓ HR of ML (ES: 0.49).
Hamacher et al. [43]	18	Endurance (cycle ergometer)	↑ RPE	Treadmill walking	Local dynamic Stability (LDS) of the walking (LyE) of 3D trunk linear acc.	↓ LDS (ES: 0.73)
Morrison et al. [21]	15 –(60–70 years—G60) 15 –(over 70 years—G70)	Endurance (incremental incline treadmill walking)	↑ RPE; ↓ Strength G70: ↓ Reaction time.	LW	Gait SP, SdL, SdD, and CAD.	G70: ↑ Gait SP, SdL, SdD and CAD
Behrens et al. [32]	16	Mental demanding (90min) vs. and control task.	↓ 10% Motivation; ↑ 100% Fatigue state	LW in ST and DT	Mean and Coeficicient of variation (CoV) of Gait Sp, SdL, StT, DsT and SwT in ST and DT condition	↑ CoV of Sp (ES: 0.66), SdL (ES: 0.67), StT (ES: 0.59), DsT (ES: 0.59) and SwT (ES: 0.41)

SL: Step Length; SdL: Stride Length; SW: Step Width; SdW: Stride Width; SD: Step Duration; SdD: Stride duration Sp: Speed; StT: Stance Time; SwT: Swing Time; DsT: Double support Time; CAD: Cadence; LW: Overground level walking; OW: Obstacle walking; acc: acceleration; vel: velocity, RPE: Rating of Perceived Exertion; var: Variability; CoV: Coefficient of variation; ML: Medial-lateral; AP: Anteroposterior; V.: Vertical, FRA: Functional Reflex Activity; DT: Dual-Task; ST: Single-Task; RW: Regular Walking; OW: Obstacle walking; LDS: Local Dynamic Stability.

### Effects of fatigue protocols on gait

Table 2 shows changes and ESs in gait outcomes after sustained muscle and mental activities. Six studies evaluated outcomes during overground walking with [25,26] or without obstruction [21,25,26,32,44,47] while single- and dual-task walking [32,44]. Five studies assessed the effects of muscle performance fatigability on gait while walking on a motorized treadmill with [27,45] or without a perturbation [23,27,43,45,46].

Muscle performance fatigability affected stride outcomes [23,25,26,44–47], gait stability [43], gait variability [44,46,47], and muscle activation [27] during gait. Stride velocity increased by ~10 cm/s (ES = 0.6), stride length by 4.8cm (ES: 0.27) [21,25,44] or by 0.3 units of normalized stride length (ES: 0.63) [23], step width by ~2 cm (ES: 0.80) [25,47], percentage of double support (~2%, ES: 1.22) [23], and a decrease in stride duration by 2ms (ES: 0.42) [25] and standard deviation of stride length by 1 cm (large ES: 0.95) [44] after sustained muscle activity. After muscle performance fatigability, local dynamic stability of 3-D trunk acceleration and symmetry in medial-lateral direction of trunk acceleration decreased by 0.1 max LyE (ES: 0.73) [43] and by 22 in harmonic rate (ES: 0.49) [46], respectively and the anteroposterior and vertical inter-stride trunk acceleration variability increased by 8% and 11% (ES: 0.8 and 2.06), respectively [47]. However, other studies did not indicate effects of muscle performance fatigability on step length (p > 0.05) [26,45,47], and unilateral muscle fatigability protocols did not affect the local dynamic stability during treadmill walking [45,46].

A decline in force induced by sustained muscle activity increased the coactivity between m. soleus and m. tibialis anterior by ~12% (ES: 0.6) and delayed functional reflex activity in the m. tibialis anterior over a 120-ms interval following treadmill decelerations by ~41% (215.7 to 174.7; ES: 0.56) [27]. While walking on an obstacle course, muscle performance fatigability reduced step duration by 5ms (ES: 0.45) and increased step velocity by 6 cm/s (ES: 0.31), step width by 1 cm (ES: 0.20), toe clearance of trailing limb to obstacle by 1 cm (ES: 0.10) [25], and the vertical loading by 4.3 N kg^-1^ m^-1^ (ES: 0.27; all p < 0.05) [26]. These results suggest that muscle performance fatigability induced adaptations in the mean and variability of spatial-temporal stride parameters during overground level walking and obstacle negotiation and increased the coactivation and delayed functional muscle reflex during treadmill walking decelerations.

Reduced mental performance was associated with an increased coefficient of variation of gait velocity from ~6% to 11%, stride length from ~4% to 7%, stance time from ~7% to 13%, double support time from ~7% to 16% (ES: 0.50 to 0.68) and swing time by from ~9% to 14% (ES: 0.41, all p < 0.05) during level walking in dual- but not in single-task condition [32].

## Discussion

We systematically reviewed studies that compared the effects of experimentally induced muscle and mental performance fatigability on gait kinematics, variability, kinetics, and muscle activity in healthy older adults. Muscle performance fatigability affects spatial and temporal features of gait and, mainly, inter-stride trunk acceleration variability. In contrast, sustained mental activity tends only to affect step variability during dual-tasking. The evidence supports the hypothesis that healthy older adults adapt spatial-temporal features of gait in a fatigue-type specific manner. We discuss these findings with a perspective on whether experimentally induced fatigue is a viable model for the study of gait adaptability in old age.

Muscle fatigue protocols were effective and induced sizable reductions in voluntary force (ES range: 0.30 to 1.32), an accepted marker of performance fatigability [8]. However, the protocols varied widely and included: 1) Repetitive muscle contractions of knee and ankle extensors with different instructions; 2) The STS task performed rapidly or at a fixed speed, and 3) Endurance tasks involving rapid walking for six minutes, incline walking on a treadmill, or incremental cycle-ergometer tests (Table 2). This large variation in methods inducing fatigue is one source of the inconsistent effects on gait because cyclical lower extremity tasks could, in fact, entrain rather than perturb gait, diminishing the interference effects and the need for participants to invoke adaptations in their walking pattern.

It is however curious that even when participants performed ~70 knee extensions or ankle plantarflexions at a maximal effort and the MVC in decreased by 50% (ES: ~1.3) [27,44], changes in spatiotemporal gait variables were moderate but in the unexpected direction (ES: 0.47 to 0.58, Table 2). Indeed, stride length (~4%), gait speed (~10%), and step width (~11%) tended to increase and stride duration (~4%) tended to decrease (ES: 0.4 to 0.8) [21,25,44]. It seems that gait has actually become more dynamic. The step and speed changes might reflect adaptations to the marked increase in trunk acceleration and variability in the vertical and anteroposterior directions (ES: 0.80 to 2.06, Table 2) [47].

Why did performance fatigability not elicit larger changes in gait and necessitate more substantive adaptive responses to the perturbations? One possibility is that torque and power demands during gait were still below the levels of joint torques and powers fatigued muscles could produce [48]. It was also reported that participants could compensate by more strongly activating muscles that were less or not affected by the task [28]. Whether gait is tested overground or on a treadmill affected the results, as studies that reported small fatigue effects on spatial-temporal parameters tested gait using an overground protocol [21,25,44,47] but those that found no effects used a treadmill [45,46]. Walking on a treadmill at a set speed makes gait kinematically uniform and minimizes the potential for adaptations to occur [49,50], especially in step variability [51,52]. This argument is borne out by a lack of fatigue effects on gait when participants were tested on treadmill [45,46] compared with the small but meaningful decreases in the autocorrelation and increases in variability of ML trunk acceleration during overground gait [47]. The use of unilateral fatigue protocols did induce some gait asymmetry but left all other gait outcomes virtually unaffected in older adults [46]. Finally, increases in gait velocity and step length after a fatigue protocol suggest that a warm-up instead of an interference effect might have occurred. However, we need to consider even these small changes in gait with caution because a number of studies reported no changes in gait metrics after a variety of muscle fatigue protocols, making all of the data combined inconsistent [26,45,46].

Muscle performance fatigability can modify muscle activation in single joint tasks and also during gait. For example, decreases in level of force delayed muscle activation onset in older adults while rising from a chair [31]. After ankle muscle fatigability, coactivation of agonist and antagonist ankle muscles increased by ~12% during gait and there was 41% delay in a functional reflex when older adults were prompted to respond to gait perturbations [27]. It is speculated that sustained muscle activity-related increase in coactivation during gait [53,54] reflects changes in the afferent feedback [29,55]. However, such an interpretation is complicated by a coupled increase in plantarflexion angular velocity and increase in coactivation of the soleus and tibialis anterior muscles during gait, a counterintuitive outcome because coactivation would tend to stiffen instead accelerate joint motion. While suggested [28], we found no direct evidence for activation substitution, i.e., reduction in muscle activation of the fatigued muscle group being compensated by increases in activation of muscles at adjacent joints. Together, the evidence is scant that there is an age- and perturbation-specific adaptation in muscle activation in response to fatigue perturbations.

Performing a mental task for a prolonged period increased gait variability only during dual-task gait [32]. This limited effect is in line with the hypothesis emerging from imaging studies suggesting the involvement of complementary brain areas in gait, attention, and executive function while walking and performing a cognitive task at the same time [36,56]. Accordingly, sustained mental activity affects cognitive functions known to be involved in gait control, resulting in an interference with gait automaticity. This interference increases step variability. In single-task conditions, the interference created by the sustained mental activity may not be large enough, producing no measurable effects on any of the gait outcomes reviewed here.

While there has been a concerted effort to use fatigue as a perturbation model (Tables 1 and 2), its viability to study the effects of age on gait adaptability remains unclear. When combined with data from young individuals, the reviewed data revealed a lack of age effect, suggesting that the nature, magnitude, and focality of the perturbations lacks specificity to age and gait. Indeed, fatigue-induced changes in gait were quantitatively similar in healthy younger and older adults and also similar in healthy older adults and Parkinsonian patients [25,28,57]. The original intent of these studies was to make healthy, fatigue-free older adults fatigued to simulate the fatigued state. However, it is unclear if the experimentally induced fatigue state and the fatigue state de novo present in older people are qualitatively and quantitatively similar. It seems that when muscle fatigue is induced with repetitive single-joint muscle contractions such as knee extension-flexion, the ensuing fatigue is predominantly a localized force impairment while the fatigue state in older adults is the result of a combination of impaired physiology, reduced homeostasis, a bias in effort perception, and altered cognitive function. When however, a multi-joint protocol is used (i.e., six-minute walk test), any adaptation in gait after the task is the result of a combined physiological and cognitive (behavioral) effect.

Such limitations and the diversity in fatigue protocols shape the implementation of this perturbation model in the future. The viability of the model will increase if studies move from its descriptive application to hypothesis-driven designs. There is a need to determine the effects of muscle performance fatigability on motor outcomes that are specific and also not specific to the fatigue task, an approach that would improve experimental control and the validity of conclusions. Future studies should also evaluate cognitive outcomes because the adaptive processes may not be confined to motor (gait) function alone. Therefore, future studies should include motor-cognitive dual-task assessments when probing age-differences in adaptations to fatigue. There is a strong need for studies examining the effects of prolonged mental tasks on gait biomechanics and variability. Such studies should set fatigability and gait as the main outcomes in older adults [58]. Such an approach would strengthen our understanding of the role cognition plays in gait control. Perhaps the most critical gap in knowledge is related to a lack of studies comparing gait outcomes in older adults with and without self-reported fatigue. Only after such studies could we meaningfully interpret gait adaptations in healthy older adults after experimentally induced muscle or mental fatigue.

In conclusion, muscle performance fatigability affects spatial and temporal features of gait and, mainly, inter-stride trunk acceleration variability. In contrast, sustained mental activity tends only to affect step variability during dual-tasking. A critical and immediate step for future studies is to determine the effects of self-reported fatigue on gait biomechanics and variability in healthy older adults to verify the viability of experimentally induced fatigue as a model for the study of gait adaptability in old age.

## Supporting information

S1 Checklist Prisma checklist. (DOC)

S1 Table Search terms. (DOCX)

S2 Table Methodological quality appraisal results. (DOCX)
