# Supplementary material for: Effects of experimentally induced fatigue on healthy older adults’ gait: A systematic review
Source: PLoS One. 2019 Dec 30;14(12):e0226939. doi: 10.1371/journal.pone.0226939 (PMC6936857; doi:10.1371/journal.pone.0226939)
Supplement: S1 Table — (DOCX) [file pone.0226939.s002.docx]

S1 Table. Search Terms

The PubMed search terms (syntax) used in the present review to identify to determine the effects of muscle and mental performance fatigability on kinematics, variability, kinetics, and muscle activity of healthy older adults’ gait. Syntax was adapted to Web of Science.

|  | Terms | Keywords |
| --- | --- | --- |
| Inclusion | #1 - Population AND | (Old OR Elderly OR Adults) |
|  | #2 - Intervention AND | (Fatigue OR Tiredness OR Fatigability) |
|  | #3 - Outcomes NOT | (Gait OR Walking OR “kinematic parameters” OR “Gait biomechanics” OR “Muscle activation” OR “Joint Coordination” OR Kinetic OR “Inverse Dynamic” OR “Gait Variability” OR “Gait Stability”) |
| Exclusion | #4 - Exclusion | (Patient NOT Disease NOT Stroke NOT Diabetes NOT Neuropathy NOT Amputation NOT "Multiple sclerosis" NOT “Cerebral palsy" NOT Parkinson NOT Cancer NOT Obese NOT Fracture NOT Dysfunction NOT “Cognitively impaired” NOT Frail NOT Demented NOT Alzheimer NOT "Pilot study") |
| Additional Filters | Language | English |
|  | Data | 1987 - 2019 |
