# Supplementary material for: Effects of experimentally induced fatigue on healthy older adults’ gait: A systematic review
Source: PLoS One. 2019 Dec 30;14(12):e0226939. doi: 10.1371/journal.pone.0226939 (PMC6936857; doi:10.1371/journal.pone.0226939)
Supplement: S2 Table — (DOCX) [file pone.0226939.s003.docx]

S2 Table. Methodological quality appraisal results. Y – yes; L - Lacking detail or clarity; N – No; AV – average. A score of one indicated high quality research and zero indicated lower quality.

| Question | Scoring criteria | Helbostad et al.^48^ | Granacher et al.^27^ | Granacher et al.^44^ | Hatton et al.^26^ | Barbieri et al.^43^ | Nagano et al. ^23^ | Toebes  et al. ^45^ | Arvir  et al. ^46^ | Hamacher et al. ^42^ | Morrison et al. ^21^ | Behrens et al. ^32^ | AV |
| --- | --- | --- | --- | --- | --- | --- | --- | --- | --- | --- | --- | --- | --- |
| 1. Research aims or questions stated clearly | Y: 1, L: 0.5, N: 0 | 1 | 1 | 1 | 1 | 1 | 1 | 1 | 1 | 1 | 1 | 1 | 1 |
| 2. Participants detailed | Number | 1 | 1 | 1 | 1 | 1 | 1 | 1 | 1 | 1 | 1 | 1 | 1 |
|  | Age | 1 | 1 | 1 | 1 | 1 | 1 | 1 | 1 | 1 | 1 | 1 | 1 |
|  | Sex | 1 | 1 | 1 | 1 | 1 | 0 | 1 | 1 | 0 | 1 | 1 | 0.8 |
|  | Height | 0 | 1 | 1 | 1 | 1 | 1 | 0 | 1 | 0 | 1 | 1 | 0.7 |
|  | Sub Total | 0.8 | 1 | 1 | 1 | 1 | 0.8 | 0.8 | 1 | 0.6 | 1 | 1 | 0.9 |
| 3. Recruitment and sampling methods described | Y: 1, L: 0.5, N: 0 | 0.5 | 0 | 0.5 | 1 | 1 | 1 | 0 | 0 | 0.5 | 1 | 0 | 0.5 |
| 4. Inclusion and exclusion criteria detailed | Y: 1, L: 0.5, N: 0 | 1 | 0.5 | 1 | 1 | 1 | 1 | 0.5 | 0.5 | 0.5 | 1 | 1 | 0.8 |
| 5. Controlled covariates | Height | 0 | 0 | 0 | 0 | 0 | 0 | 0 | 0 | 0 | 0 | 1 | 0.1 |
|  | Gait Speed | 1 | 0 | 1 | 0 | 0 | 1 | 1 | 0 | 0 | 0 | 0 | 0.4 |
|  | Age | 1 | 1 | 1 | 1 | 1 | 1 | 1 | 1 | 1 | 1 | 1 | 1 |
|  | Gender | 0 | 1 | 1 | 0 | 1 | 0 | 0 | 0 | 0 | 1 | 0 | 0.4 |
|  | Asymmetry | 0 | 1 | 1 | 1 | 0.5 | 1 | 1 | 1 | 0 | 0 | 0 | 0.6 |
|  | Strength | 0 | 1 | 1 | 1 | 1 | 1 | 1 | 0 | 0 | 1 | 0 | 0.6 |
|  | Sub Total | 0.3 | 0.7 | 0.8 | 0.5 | 0.6 | 0.7 | 0.7 | 0.3 | 0.2 | 0.5 | 0.3 | 0.5 |
| 6. Key outcome variables clearly described | Y: 1, L: 0.5, N: 0 | 1 | 1 | 1 | 1 | 1 | 1 | 1 | 1 | 1 | 1 | 1 | 1 |
| 7. Adequate methodology able to repeat study | Participants | 1 | 1 | 1 | 1 | 1 | 1 | 1 | 1 | 1 | 1 | 1 | 1 |
|  | Equipment | 1 | 1 | 1 | 1 | 1 | 1 | 1 | 1 | 1 | 1 | 1 | 1 |
|  | Procedure | 1 | 1 | 1 | 1 | 1 | 1 | 1 | 1 | 0 | 1 | 1 | 0.9 |
|  | Processing | 1 | 1 | 1 | 1 | 1 | 1 | 1 | 1 | 1 | 1 | 1 | 1 |
|  | Statistics | 1 | 1 | 1 | 1 | 1 | 1 | 1 | 1 | 1 | 1 | 1 | 1 |
|  | Sub total | 1 | 1 | 1 | 1 | 1 | 1 | 1 | 1 | 0.8 | 1 | 1 | 1 |

| 8. Methodology able to answer research question | Participants | 1 | 1 | 1 | 1 | 1 | 1 | 1 | 1 | 1 | 1 | 1 | 1 |
| --- | --- | --- | --- | --- | --- | --- | --- | --- | --- | --- | --- | --- | --- |
|  | Equipment | 1 | 1 | 1 | 1 | 1 | 1 | 1 | 1 | 1 | 1 | 1 | 1 |
|  | Procedure | 1 | 1 | 1 | 1 | 1 | 1 | 1 | 1 | 1 | 1 | 1 | 1 |
|  | Processing | 1 | 1 | 1 | 1 | 1 | 1 | 1 | 1 | 1 | 1 | 1 | 1 |
|  | Statistics | 1 | 1 | 1 | 1 | 1 | 1 | 1 | 1 | 1 | 1 | 1 | 1 |
|  | Sub total | 1 | 1 | 1 | 1 | 1 | 1 | 1 | 1 | 1 | 1 | 1 | 1 |
| 9. Reliability of the methodology stated | Y: 1, N: 0 | 0 | 0 | 0 | 0 | 0 | 0 | 0 | 0 | 0 | 0 | 0 | 0.0 |
| 10. Internal validity of the methodology stated | Y: 1, N: 0 | 0 | 0 | 0 | 0 | 0 | 0 | 0 | 0 | 0 | 0 | 0 | 0.0 |
| 11 Research questions answered adequately in the discussion | Y: 1, N: 0 | 1 | 1 | 1 | 1 | 1 | 1 | 1 | 1 | 1 | 1 | 1 | 1 |
| 12. Key ﬁndings supported by the results | Y: 1, N: 0 | 1 | 1 | 1 | 1 | 1 | 1 | 1 | 1 | 1 | 1 | 1 | 1 |
| 13. Key ﬁndings interpreted in a logical manner which is supported by references | Y: 1, N: 0 | 1 | 1 | 1 | 1 | 1 | 1 | 1 | 1 | 1 | 1 | 1 | 1 |
| 14. Clinical implications stated | Y: 1, L: 0.5, N: 0 | 1 | 1 | 1 | 1 | 1 | 1 | 1 | 1 | 1 | 1 | 1 | 1 |
